# Organizational and training factors that promote team science: A qualitative analysis and application of theory to the National Institutes of Health’s BIRCWH career development program

**DOI:** 10.1017/cts.2016.17

**Published:** 2017-02-08

**Authors:** Jeanne-Marie Guise, Susan Winter, Stephen M. Fiore, Judith G. Regensteiner, Joan Nagel

**Affiliations:** 1 Department of Obstetrics and Gynecology, Division of Maternal Fetal Medicine, OHSU School of Medicine, Portland, OR, USA; 2 Department of Medical Informatics and Clinical Epidemiology, OHSU School of Medicine, Portland, OR, USA; 3 Department of Emergency Medicine, OHSU School of Medicine, Portland, OR, USA; 4 OHSU-PSU School of Public Health, Portland, OR, USA; 5 College of Information Studies, University of Maryland, College Park, MD, USA; 6 Cognitive Sciences Program, Department of Philosophy, University of Central Florida, Orlando, FL, USA; 7 Center for Women’s Health Research, Division of General Internal Medicine and Cardiology, University of Colorado School of Medicine, Aurora, CO, USA; 8 Center for Advancing Translational Sciences, National Institutes of Health, Bethesda, MD, USA

**Keywords:** Organizational models, research, models, translational medical research, interdisciplinary communication

## Abstract

**Introduction:**

Research organizations face challenges in creating infrastructures that cultivates and sustains interdisciplinary team science. The objective of this paper is to identify structural elements of organizations and training that promote team science.

**Methods:**

We qualitatively analyzed the National Institutes of Health’s Building Interdisciplinary Research Careers in Women’s Health, K12 using organizational psychology and team science theories to identify organizational design factors for successful team science and training.

**Principal Results:**

Seven key design elements support team science: (1) semiformal meta-organizational structure, (2) shared context and goals, (3) formal evaluation processes, (4) meetings to promote communication, (5) role clarity in mentoring, (6) building interpersonal competencies among faculty and trainees, and (7) designing promotion and tenure and other organizational processes to support interdisciplinary team science.

**Conclusion:**

This application of theory to a long-standing and successful program provides important foundational elements for programs and institutions to consider in promoting team science.

## Introduction

Health and well-being, education and learning, climate change, and disaster response are just a few examples of the complex domains that the modern scientific enterprise is asked to address. The complexity of these societal problem calls for large-scale, sustained, collaborative, and interdisciplinary approaches. Governments, charitable foundations, and for-profit companies are investing in scientific collaborations that cut across all types of science, fueling the demand for integrated interdisciplinary perspectives [1]. The centrality of interdisciplinarity to innovation has been widely acknowledged [2]. It has been reported that important discoveries and outcomes emerging from cutting-edge interdisciplinary research have fueled economic growth and improved societal vitality [3, 4].

Expanding innovative interdisciplinary science requires changes in institutions that traditionally have focused on individual scholarship including universities and funding agencies. In addition, changes in the approaches and attitudes of scholars themselves will be necessary. In the long term, institutional systems and processes of promotion, and peer review of grants and manuscripts must include a broader perspective that encompasses the scholarly efforts of interdisciplinary science collaborations. At present, the individualistic focus of traditional scientific training is ill-suited to meet the demand for interdisciplinary scientists [5]. A number of programs have been created to encourage institutions to develop collaborative, interdisciplinary scientists, but this change needs to become widespread [6].

Empirical research has shown that young scholars are particularly attracted to interdisciplinary research, especially for the opportunity to work on important problems and collaborate with others [7]. Developing interdisciplinary researchers requires educational experiences, exposing scientists to interdisciplinary environments, and developing deep knowledge of multiple fields. Further, scientists need to develop the skills required to become effective members of interdisciplinary collaborations and leaders for institutional change [8]. The nature of these skills is not yet fully understood and efforts to develop interdisciplinary researchers have been limited [9]. In addition, there is growing concern that graduates of many interdisciplinary programs face significant disadvantages in their careers including difficulty in finding interdisciplinary positions, funding, and publication outlets appropriate for their cross-cutting areas of research [6, 7]. This can negatively impact professional development, resulting in a career pipeline that loses a large fraction of scientific talent at each career stage [10].

The objective of this study is to apply organizational and psychological sciences theory to evaluate one of the longest standing programs for training interdisciplinary team scientists [the US National Institutes of Health (NIH) Office of Research on Women’s Health (ORWH) Building Interdisciplinary Research Careers in Women’s Health (BIRCWH) Program (NIH ORWH BIRCWH K12 Program)] to understand the structure of scholarly institutions and the training that promotes team science.

## Methods

### Program Analysis Applying Organizational and Social Theory

Programs can be analyzed through the lens of organizational and social science theory. We briefly review recent developments in the conduct of science, highlighting the importance of complex organizations such as universities and interdisciplinary collaborative teams. Drawing on theory and concepts from organizational and social sciences, 1 qualitative expert trained in organizational studies and 1 BIRCWH Director experienced in qualitative research analyzed the BIRCWH program to identify mechanisms underlying its success and the implications for mutually informed theory and practice. To ensure reliability of the coding structure, all themes were reviewed with the larger multidisciplinary research team, which included a qualitative expert trained in team science, a BIRCWH Director, and a national BIRCWH Program leader representing a major governmental funder. Discrepancies between coders were resolved through discussion in the larger research team. Finally, we integrate theory and practice, identifying additional areas for research, and set the stage for translating the success of this program to support other complex interdisciplinary research endeavors.

## Results

### Developments in the Conduct of Team Science

Teams are defined as “two or more individuals who must interact and adapt to achieve specified, shared, and valued objectives” [11]. They rely on multiple information sources and intensive communication and have clearly demarcated roles. Members hold task-relevant knowledge with meaningful task interdependencies and must coordinate their actions and interactions so that goals and objectives are met. Teamwork allows people to achieve objectives that an individual could not achieve alone while maintaining only partially overlapping knowledge [12].

Teamwork in science varies in complexity based upon a variety of factors, including the number of scientific and professional disciplines involved. Science of Team Science scholars have defined the various ways that science teams interact to produce knowledge [1, 13–17]. At the simplest level, multidisciplinary research brings scientists together to accomplish a broader analysis of a problem. This team may periodically meet, but participants work largely independently and their contributions are more complementary than integrative. Interdisciplinary research involves the systematic integration of information, data, techniques, tools, perspectives, concepts, and/or theories from multiple bodies of specialized knowledge [15] to develop something new that advances fundamental understanding and solves problems that a single discipline could not [1]. Transdisciplinary research brings a multi-level perspective to problems (eg, from cellular to societal) and may include partners from outside science (eg, patients, families, and advocacy groups) as active participants to develop a comprehensive understanding of complex societal challenges and accelerate the translation to application.

### Description of the BIRCWH K12 Program

The NIH ORWH BIRCWH K12 Program provides protected time for research and salary support to early career clinical and basic scientists to conduct research in women’s health or sex differences research, and provides each participant an interdisciplinary team of mentors to guide them in building an interdisciplinary career.

**Fig. 1:**
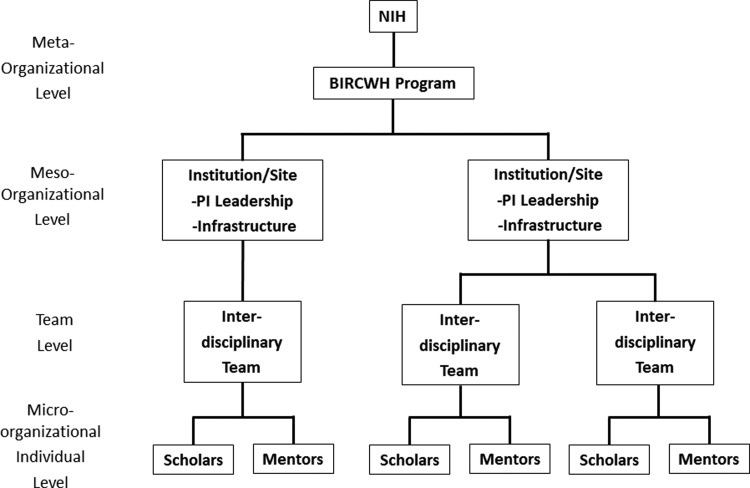
Organizational structure of the Building Interdisciplinary Research Careers in Women’s Health (BIRCWH) according to the Science of Team Science (SciTS) multi-level framework. NIH, National Institutes of Health.

The ORWH has long recognized that “the study of women’s health across the lifespan requires an interdisciplinary approach to research, bridging basic and clinical science, and incorporating new models of collaboration, institutional support, and ways of evaluating those who conduct it” [18]. Reports of a dearth of data on women’s health conditions and a need to support and protect time for women’s health research, were the impetus for the ORWH to establish the BIRCWH K12 Program to specifically encourage and support development of interdisciplinary team science on women’s health and gender topics [19–22]. Fig. 1 provides an overview of the program’s structure. The program is designed around 3 pillars: (1) career development, (2) mentoring, and (3) interdisciplinary research. Although there are differences in the scientific focus and administrative details across participating institutions, all provide 75% protected time for scholars to pursue mentored research in women’s health, require interdisciplinary team mentoring, and have shared investments between NIH and the institution. The BIRCWH requires that the principal investigator (PI) of a program is a high-ranking leader in the institution, “such as a Dean, Department Chair, or Director of a research center or interdisciplinary institute” [18], specifically to ensure the integration of team science into the fabric of the institution.

Published evaluations provide evidence of the impact of the BIRCWH program since 2000. In October 2013, 27 sites were currently active and 77 funding awards had enabled 542 BIRCWH scholars to participate at 39 institutions. A majority of BIRCWH scholars had applied for competitive grants after completing training and approximately two-thirds were successful [23]. Scholars participating in the BIRCWH were also found to have a grant success rate of 38% compared with the NIH overall average of 29%.

### Application of Organizational and Psychological Sciences Theory to Evaluate the BIRCWH

The application of theory and concepts from research on organizations and teams applied to the NIH ORWH BIRCWH K12 Program identified themes for the design of institutions and programs to support team science and the development of interdisciplinary team scientists. Seven themes for infrastructural elements that promote team science were common across BIRCWH programs.

#### Semiformal Meta-Organizational Structure

Designing scientific research organizations is challenging [24]. A multi-level mixed method approach has been used to understand the Science of Team Science [25]. Applying this model to the BIRCWH program reveals important insights on how the semiformal organizational structure of the BIRCWH addresses challenges faced by investigators and institutions in conducting and supporting team science (Fig. 1).

An important factor in the BIRCWH program’s success is its complex innovative cross-organizational structure that allows it to leverage existing resources by, for example, requiring shared NIH and institutional investments, thereby maximizing its impact, flexibility, and longevity (macro-level and meso-level organization). The BIRCWH program spans multiple academic research institutions (meso-level), allowing the program to leverage local facilities, investigator knowledge, and staff while being supported through funding and coordination through a Federal agency (NIH) (meta-level). This structure allows the BIRCWH program to act as a bridge that crosses research institutions and the Federal Government coordinating expertise that is widely shared across its member organizations instead of centralized and isolated within the BIRCWH senior staff. Table 1 provides a description of each organizational level. Organizational scientists would consider this kind of semiformal organization [26], an important structure for developing and maintaining communities of practice that link people who perform the same kind of work but at different locations [27]. Consistent with organizational research, this structure provides the flexibility that is crucial to success allowing sites to tailor activities to fit local circumstances [28]. For instance, each BIRCWH program has different specific offerings for their BIRCWH Scholars with regard to, for example, training sessions and skill development, while all have the common goal of developing research careers [29].

**Table 1:** Multi-level mixed method analysis of strategies that promote collaborative team science

Level	Challenges	Collaboration mechanisms	Advantages
Macro: NIH-BIRCWH	Leveraging limited scope and resources	Semiformal organizational structure Institution-level requirements (eg, protected time for mentoring, interdisciplinary teams, shared investments, high-ranking PI)	Large scale Consistent Flexibility/customization Institution-wide change Cost efficiencies
Meso: Institutions/sites	Integrating institution-wide change Compatible mental models Coordination Human resources Knowledge flows Standardized work routines	Promotion and tenure criteria Focus, knowledge, goals, experience, domain In-person program-wide meetings Formal written progress reports Workshops/seminars Mentor and scholar team meetings required Designated mentor roles	Recognition of interdisciplinary researchers and Mentors Women’s health, health care domain, interdisciplinary experience Enhanced knowledge Networking Continuous improvement Coordinated activities
Teams	Coordination	Research symposium Meeting of Directors Workshops Presentations Speed mentoring	Multiple compatible mental models (equipment, task, team interaction, teammate)
Scholars	Interpersonal competencies Relationship management Intellectual orientation	Research fora, clubs, societies	Active listening Interdisciplinary appreciation
Mentors		Mentor training roles: career vs. science Frequent meetings Written contracts Networking ability Formal assessments	Enhanced knowledge

NIH, National Institutes of Health; BIRCWH, Building Interdisciplinary Research Careers in Women’s Health; PIs, Principal Investigators.

#### Shared Research Context and Goals

Coordinating the work of multiple individuals increases in difficulty as the number of people increases and their knowledge diverges [24]. This includes not only scientific knowledge associated with their research domain, but also more practical/logistic knowledge. Simply put, 2 people in the same program, building, research institution, and state will have more shared knowledge than will 2 people in different programs. Although it may seem inconsequential, even a rudimentary lack of shared knowledge creates coordination problems. For example, people in different locations may not know about important features such as traffic delays affecting a member’s ability or availability.

The BIRCWH program has been remarkable in overcoming the many challenges associated with coordinating activities that are distributed across geography, time zones, and scholarly domains. Understanding the kinds of shared factors that ease coordination and those that present significant challenges and the mechanisms used to overcome them can facilitate their translation to other research communities. First, the nature of the work conducted across BIRCWH centers provides a *shared research problem (women’s health and gender research)* that is an important mechanism to overcome coordination barriers [30]. Second, there is *a shared research context (academic research organizations)*. The BIRCWH program benefits from the fact that all of its participants work in the health area so they share knowledge about this area in general and about how health and healthcare research organizations operate. Third, there are *shared goals* around professional development and mentoring goals and have shared experiences with regard to how the centers are evaluated (eg, scholars, program level). Finally, there is the more general *shared domain* [28].

#### Formal Evaluation Processes

The scale of the BIRCWH program is too large for informal coordination among the BIRCWH Directors. Because of this, formal coordinating processes and practices such as performance evaluations, program evaluations, and an annual national meeting sponsored by the ORWH have been created [31]. To minimize the administrative and reporting challenges, principal investigators and scholars work with NIH to evaluate strategies and identify best practices that can overcome challenges researchers may face resulting in a cycle of continuous improvement [29, 32]. The BIRCWH PIs have published on best practices and what works and what does not work for team science [29, 32]. In addition, ORWH has published evaluations in its annual report to the NIH, Advisory Committee for Women’s Health Research and is in the process of conducting an evaluation of the BIRCWH program over the last decade.

#### Meetings to Promote Communication

Although time consuming, meetings are acknowledged as an important coordination mechanism in collaborative science and are particularly important in the BIRCWH program (Table 2).

**Table 2:** Building Interdisciplinary Research Careers in Women’s Health (BIRCWH) meeting types and purposes

Meeting type/name	Key elements/items covered
Macro-level meetings—meetings represent field-level research issues as well as national BIRCWH program-level issues. Meetings are held annually	
Annual NIH Interdisciplinary Research Symposium	Showcase research findings from BIRCWH scholars Discuss key methodologic issues affecting field
Annual BIRCWH Meeting	Support coordination of resources across network Discuss equipment and facilities available within network Discuss best practices and work routines Provide opportunity for knowledge transfer and community building among BIRCWH investigators and scholars
Annual BIRCWH Meeting	Support coordination of resources across network Discuss equipment and facilities available within network Discuss best practices and work routines Provide opportunity for knowledge transfer and community building among BIRCWH investigators and scholars
Micro-level meetings—meetings help coordinate activities and knowledge at the local level. Frequency of meetings vary by local site	
Research Meetings	Traditional project meetings (for mentors and participants) around research program needs
Mentoring Meetings	Individualized meetings for mentees with specific mentors based upon identified roles (eg, primary mentor, secondary mentor)
Interdisciplinary Team Mentoring Meetings	Complete mentor team meetings held 2–4 times per year to develop common knowledge and ensure shared goals are being met
Site Mentor-Mentee Meetings	Idiosyncratic team meetings to bring together scholars, mentors, or PI/PDs, dependent upon project needs and goals; these can include: Weekly meetings for the primary mentor and the scholar Biweekly meetings that focus on the interdisciplinary science challenges Bimonthly meetings that address professional issues
Prior Meetings	Meetings for faculty on independent or center-based K programs to share/discuss research

NIH, National Institutes of Health; PIs, Principal Investigators.

The BIRCWH has instituted a regular annual in-person national meeting among the BIRCWH principal investigators and scholars to allow ongoing coordination, and sharing of best practices that programs can use to design their local programs and meetings. At a local level, many BIRCWH sites have divided mentoring according to roles (eg, primary mentor, secondary mentor) to promote efficiencies and minimize conflict across mentors. In addition to one-on-one mentor meetings, programs also promote regular in-person meetings with the scholar’s entire mentoring team.

The knowledge and social capital of mentors, directors, and scholars are augmented with seminars, workshops, a virtual mentoring network, and the annual national BIRCWH meeting [29]. In addition to facilitating coordination, meetings are powerful organizational mechanisms that support knowledge sharing, networking, and collaboration among BIRCWH investigators and scholars, at local, regional, and national levels.

Design choices that affect knowledge flows are another important aspect of science organizations and both the BIRCWH program and the sites have developed policies that specify the timing, methods, and content of these knowledge flows [24]. Here, again, meetings can play an important role and much of the mentoring takes place during face-to-face meetings. Importantly, this has been found to be the best communication method when there is a need to reduce ambiguity or equivocality [24]. At a local level, BIRCWH programs hold various types of meetings including gatherings for BIRCWH scholars from disparate fields to present their research progress and receive feedback and clubs/societies such as K clubs/Socrates society that bring together junior faculty scholars from other career development programs such as the Clinical and Translational Science Awards to learn from each other. In this way, BIRCWH bridges scientific disciplines and fields and fosters cross-fertilization and exposure to variables that could impact the research question being asked.

Further, in order to ensure there is common ground on what is discussed, written communication is used to clarify expectations through email, agreement forms, and career development plans. This provides a permanent record, is better suited to handling more complex information, and can be used to guide future action.

#### Role Clarity in Mentoring

Mentoring is the second pillar of the BIRCWH program. Mentoring in science has traditionally cultivated independence and intellectual growth within a narrow field of inquiry. Interdisciplinary research mentoring should also cultivate mentees’ skills for managing team interactions and help identify training needs relevant to mentee roles within a team [1]. For example, helping mentees understand the roles they play within a functional unit and the larger organizational context or helping mentees understand and address their weaknesses. Detailed analyses of the BIRCWH interdisciplinary mentoring program showed that clear roles should be outlined (eg, primary, career issues, or scientific content mentors). A primary mentor and mentee should meet frequently and written contracts should be used to manage expectations [29]. The networking ability of the mentor is also critical in promoting interdisciplinary science, so mentors need to have strong collaborative skills and the ability to help the mentee build new collaborative relationships within and outside of their discipline and organization. To ensure continual improvement, many BIRCWH sites include formal assessments of mentors’ self-efficacy in mentoring. In addition, the majority of sites conduct assessments/evaluations of how well the mentor-mentee relationship is working and provide training to develop mentoring skills [29].

#### Building Interpersonal Competencies Among Faculty and Trainees

Developing interpersonal competencies that support collaborative work [33] and the intellectual orientation to manage the challenges of crossing disciplinary boundaries [34] are foundational to team success. Interdisciplinary teams perform better when their members are motivated to acquire knowledge from other disciplines that are relevant to scientific problem solving and the skills and knowledge that are critical to synthesize varied concepts and theories. Interpersonal competencies including communication competencies advance by improving communication effectiveness and enhancing identification “with the collaborative and integrative goals of the team” [34]. The design of the BIRCWH program cultivates these competencies scholars in that they are interfacing with multiple mentors, organizing meetings and coordinating feedback from multiple perspectives; working with interdisciplinary staff given their multi-faceted research; and working with other scholars in their program and nationally from other scientific domains.

#### Designing Promotion and Tenure and Other Organizational Processes to Support Interdisciplinary Team Science

Interdisciplinary research is one of the critical pillars of the BIRCWH program. Efforts to recognize and reward interdisciplinary research both at the national and institutional level have been made by BIRCWH programs at many sites. For example, the University of Colorado and Oregon Health & Science University (OHSU) promotion and tenure processes include explicit statements recognizing the value of interdisciplinary and collaborative science and setting out procedures for documenting team members’ individual contributions: “…additional evidence should also be provided such as letters from the PIs or research group heads with whom you have collaborated, outlining in detail your specific contributions and the unique skills that you brought to the team” (University of Colorado), and faculty are encouraged to demonstrate “leadership and innovation in contributions to collaborative research efforts” (OHSU). Similarly, BIRCWH programs contributed to national statements and processes supporting team science such as this contribution of the University of North Carolina’s BIRCWH to NIH policies: “biomedical science is placing more and more emphasis on interdisciplinary team activities. Therefore, when relevant, a faculty member’s contributions to interdisciplinary teamwork will be given careful consideration” [35]. These changes demonstrate the value and impact of programs that ignite a culture of collaborative interdisciplinary team science across academic organizations and science at a national level. Efforts to address promotion and tenure are ongoing and has been a major theme of National BIRCWH PI meetings.

## Discussion

Examining the successes of the NIH ORWH BIRCWH K12 Program in promoting team science suggests 7 mechanisms for institutions and programs to support team science: (1) semiformal meta-organizational structure, (2) shared context and goals, (3) formal evaluation processes, (4) meetings to promote communication, (5) role clarity in mentoring, (6) building interpersonal competencies among faculty and trainees, and (7) designing promotion and tenure and other organizational processes to support interdisciplinary team science. Strategic choices in the design and implementation of the BIRCWH program have contributed to its track record of success. It represents a semiformal organization that spans institutions and leverages their resources to create a community of practice focused on improving women’s health through interdisciplinary research. With considerable decision making and control remaining in the hands of the individual sites, the NIH ORWH is able to play a predominantly coordinating role for the BIRCWH program standardizing core elements and encouraging team mentoring and co-support while enabling flexibility in the implementation details like the size and roles of the mentoring teams. This structure enhances the value of the meetings that are critical to coordination, dissemination of information, team mentoring, and career development.

Although the BIRCWH program has been successful in nurturing interdisciplinary science, the transformation in women’s health is not complete. A full acceptance of interdisciplinary team science will require continued changes in norms, values, and expectations that may take decades to unfold. Methods for conceptualizing and evaluating the quality of a scientific collaborative practice as a whole may need to be created, shifting our focus to the cultivation of scholars who stay active in a domain such as women’s health in an interdisciplinary fashion (rather than siloed) and move into leadership positions over time. Understanding, appreciating, and evaluating the multiple contributions members make to team success will require metrics that encompass a variety of team roles as well as accommodate team members who may specialize in a single area. Clearly, traditional technologies (eg, computers and cell phones) still feature prominently in communications (eg, email, teleconferences); however, additional attention is being given to use of webinars, videoconferencing, Wikis, YouTube channels, blogs, and tweets for training and/or connecting individuals, team, and programs. In order to extend theory and practice for enduring semiformal organizational structures, it may be helpful for BIRCWH Programs and others to examine the best use of technology across sites to inform best uses and ease adoption [24, 36].

### Limitations

Little is known about how to design effective interdisciplinary science enterprises; the NIH ORWH BIRCWH K12 Program represents an important model for organizational and team science. Although the BIRCWH program represents a long experience of large research network, it is only one example. More studies are needed to understand whether the experience of the BIRCWH holds true across other settings and programs. Furthermore, as science becomes increasingly interdisciplinary, it is unclear whether developing individuals with interdisciplinary skills, interdisciplinary teams, or both will be most effective. Future efforts and research should help identify the most effective organizational structures and infrastructures that support and incentivize interdisciplinary training, successful mentoring, and interdisciplinary teams.
